# Beyond socioeconomic status: structural determinants and strategic approaches to regional variation in left ventricular assist device utilization

**DOI:** 10.1007/s12471-026-02047-5

**Published:** 2026-05-08

**Authors:** Toshihide Izumida, Teruhiko Imamura

**Affiliations:** Second Department of Internal Medicine, University of Toyama, 930-0194 Toyama, Japan

We have read with great interest the manuscript, which demonstrates that significant regional differences in left ventricular assist device (LVAD) utilization persist despite the absence of socioeconomic disparities and geographic distance effects [1]. Beyond referral awareness and timing, constraints in multidisciplinary manpower and institutional capacity at LVAD centers may limit patient evaluation, implantation volume, and longitudinal follow-up, thereby contributing to regional variation.

In Japan, where universal healthcare coverage similarly reduces financial barriers, regional disparities have been addressed primarily through locally driven, institution-led efforts rather than centralized national policies. For instance, academic centers and regional networks have developed structured education programs, such as the BIND initiative, [2] as well as shared-care frameworks exemplified by the *Kyushu style*, which clearly delineate responsibilities between implanting and referring hospitals [3].

Given that LVAD implantation in the Netherlands is concentrated in four centers, we would appreciate clarification regarding whether national educational programs, standardized referral pathways, or formal shared-care models have been implemented to harmonize access across regions. Furthermore, how do implantation centers coordinate outreach, referral processes, and capacity management across regions? Clarification of these policy-level and institutional strategies would help contextualize the observed regional variation.

## References

[1] Drost VCE, Wösten M, Otterspoor LC, et al. Left ventricular assist device utilization across the different regions of the Netherlands. Neth Heart J. 2026; 10.1007/s12471-026-02019-9. 41665862 10.1007/s12471-026-02019-9 PMC12920953

[2] Ichihara Y, Niinami H. Mission as a Heart Transplantation Institute in Japan:Role of Tokyo Women’s Medical University in Development of Advanced Heart Failure Therapy. Kyobu Geka. 2022;75(1):4–9. 35249070

[3] Ushijima T, Fujino T, Komman H, et al. Kyushu-style collaboration between the implantable ventricular assist device implantation and management centers: a republication of the article published in Japanese journal of artificial organs. J Artif Organs. 2024;27(3):198–202. 10.1007/s10047-024-01451-1. 38879833 10.1007/s10047-024-01451-1

